# Supplementary material for: Examination of Prognostic Factors Affecting Long-Term Survival of Patients with Stage 3/4 Gallbladder Cancer without Distant Metastasis
Source: Cancers (Basel). 2020 Jul 27;12(8):2073. doi: 10.3390/cancers12082073 (PMC7464443; doi:10.3390/cancers12082073)
Supplement: Supplementary file 1 [file cancers-12-02073-s001.pdf]

cancers MDPI

Examination of Prognostic Factors Affecting Long-Term Survival of Patients with Stage 3/4 Gallbladder Cancer without Distant Metastasis

Ryota Higuchi, Takehisa Yazawa, Shuichirou Uemura, Yutaro Matsunaga, Takehiro Ota, Tatsuo Araida, Toru Furukawa and Masakazu Yamamoto

Table S1. (**A**) Number at risk, median survival time, and 5-year survival rate based on degree of liver invasion. (**B**) Number at risk, median survival time, and 5-year survival rate based on degree of hepatoduodenal ligament invasion.

(A)					
**Liver Invasion**	**Number at Risk**			**Median Survival Time (Months)**	**5-Year Overall Survival (%)**
	**0 M**	**24 M**	**60 M**		
Hinf0; no invasion	76	52	33	56	48
Hinf1; Direct invasion of the liver parenchyma, <5 mm	27	9	5	28	40
Hinf2; Direct invasion of the liver parenchyma, ≥5 mm, <20 mm	26	11	4	16	23
Hinf3; Direct invasion to the liver parenchyma, ≥20 mm	28	7	3	18	14

(B)					
**Hepatoduodenal Ligament Invasion**	**Number at Risk**			**Median Survival Time (Months)**	**5-Year Overall Survival (%)**
	**0 M**	**24 M**	**60 M**		
Binf0; no invasion	80	52	33	56	49
Binf1; Invasion of the right margin of the hepatoduodenal ligament, but not of the left margin	24	9	5	20	41
Binf2; Invasion of the left margin of the hepatoduodenal ligament, but not of the entire ligament	37	11	4	17	16
Binf3; Invasion through the hepatoduodenal ligaments	16	7	3	16	19

Table S2. (**A**) Number at risk, median survival time, and 5-year survival rate based on the number of prognostic factors predicted preoperatively in patients with stage 3/4 gallbladder cancer without distant metastases. (**B**) Number at risk, median survival time to recurrence, and 5-year recurrence-free survival rate based on the number of prognostic factors predicted preoperatively in patients with stage 3/4 gallbladder cancer without distant metastases.

(A)					
**Number of Risk Factor**	**Number at Risk**			**Median Survival Time (month)**	**5-Year Overall Survival Rate (%)**
	**0 M**	**24 M**	**60 M**		
Zero factors	68	45	31	NA	54.2
One factor	54	28	13	31	34.2
Two factors	27	5	1	15	5.87
Three factors	8	1	0	8.5	0

(B)					
**Number of Risk Factor**	**Number at Risk**			**Number at Risk**	**Number at Risk**
	**0 M**	**24 M**	**60 M**		
Zero factors	68	42	27	46	47.4
One factor	54	24	11	22	23.6
Two factors	27	3	1	9	10.0
Three factors	8	0	0	7	0

Table S3. Characteristics of cases.

**Total**	***n* = 157**
Period 1985–1999/2000–2017	41% / 59%
Age, median (minimum-maximum)	69 (31–88)
Female	55%
Incidental gallbladder cancer	10%
Cholecystectomy/Liver bed resection/S4aS5/≥2 sections	23%/21%/27%/29%
With/without bile duct resection/pancreatoduodenectomy	50%/25%/24%

Vascular reconstruction (portal vein/hepatic artery)		16% (15%/1%)
Blood loss, median (minimum-maximum)		1000 (5–9200)
Surgery time, median (minimum-maximum)		345 (90–740)
R0 resection		80%
AJCC stage (IIIA/IIIB/IVA/IVB)		33 (21%) /68 (43%) /31(20%) /25 (16%)
Morbidity (Clavien-Dindo classification ≥3)		41%
Surgical mortality		4.5% (7/157, details of each case follow)
1987	Right hepatectomy with BDR	Biliary fistula, liver failure
1988	Right HLPD, HA, PV	Bleeding, liver failure
1990	Right HLPD, HA, PV	Liver stump abscess, liver failure
2001	Right hepatectomy with BDR	Liver stump abscess, liver failure
2005	Right trisectionectomy, BDR, PV	Liver stump abscess, liver failure
2006	Right hepatectomy with BDR	Liver stump abscess, liver failure
2015	Liver bed resection with lymph node dissection	Lymph fistula, liver cirrhosis

AJCC, American Joint Committee Classification ; BDR, bile duct resection; HLPD, hepato-ligament-pancreaticoduodenectomy; HA, hepatic artery resection: PV, portal vein resection.

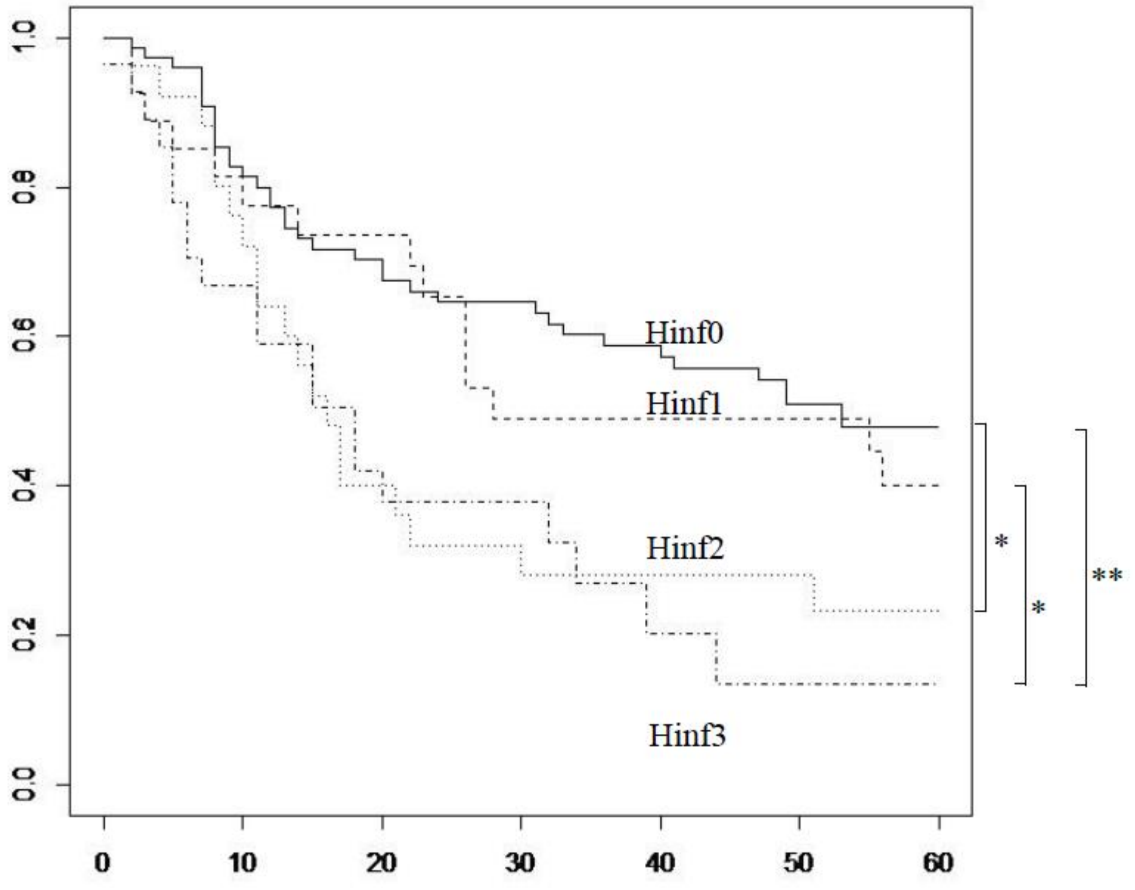

(**A**)

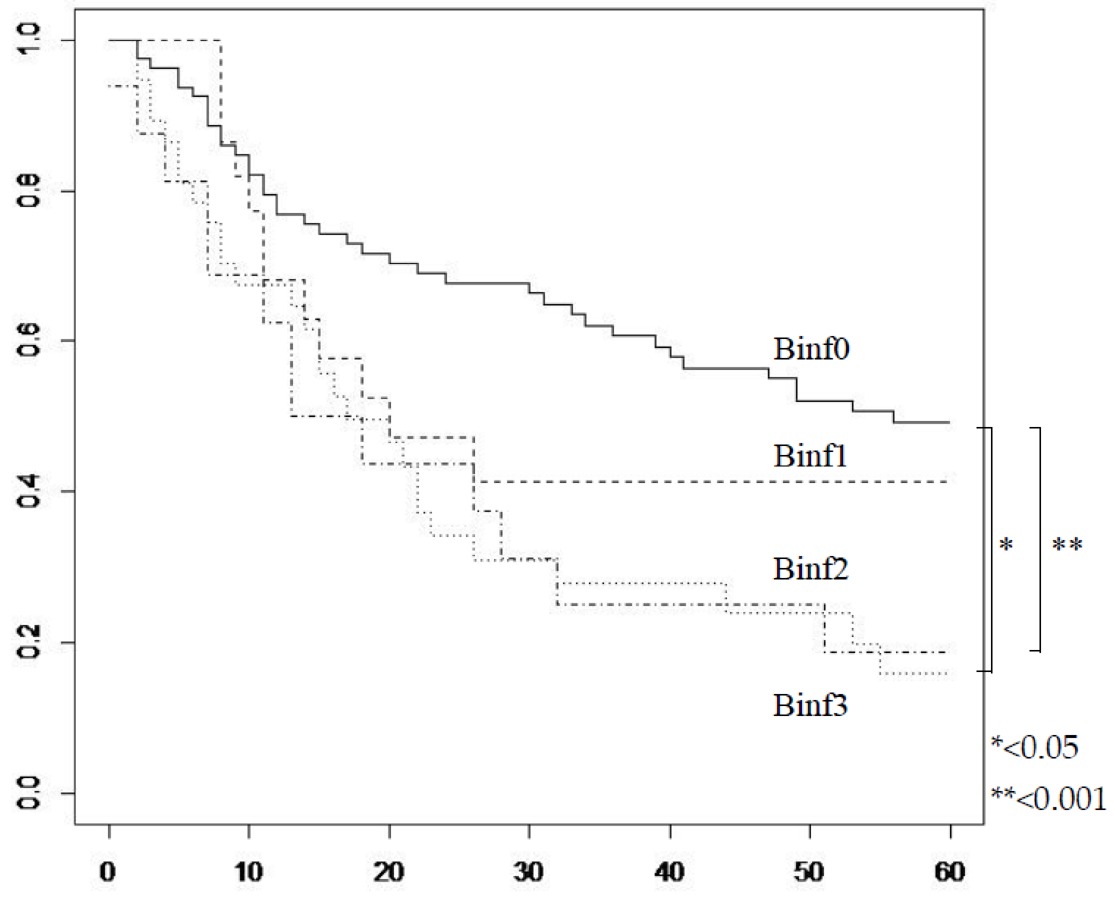

(**B**)

Figure S1. (**A**) Overall survival rate based on degree of liver invasion. Hinf0, no invasion; Hinf1, Direct invasion of the liver parenchyma, <5 mm; Hinf2, Direct invasion of the liver parenchyma, ≥5 mm, <20 mm; Hinf3, Direct invasion to the liver parenchyma, ≥20 mm. *< 0.05, **< 0.01. (**B**)**.** Overall survival rate based on degree of hepatoduodenal ligament invasion. Binf0, no invasion; Binf1, Invasion of the right margin of the hepatoduodenal ligament, but not of the left margin; Binf2, Invasion of the left margin of the hepatoduodenal ligament, but not of the entire ligament; Binf3, Invasion through the hepatoduodenal ligaments.

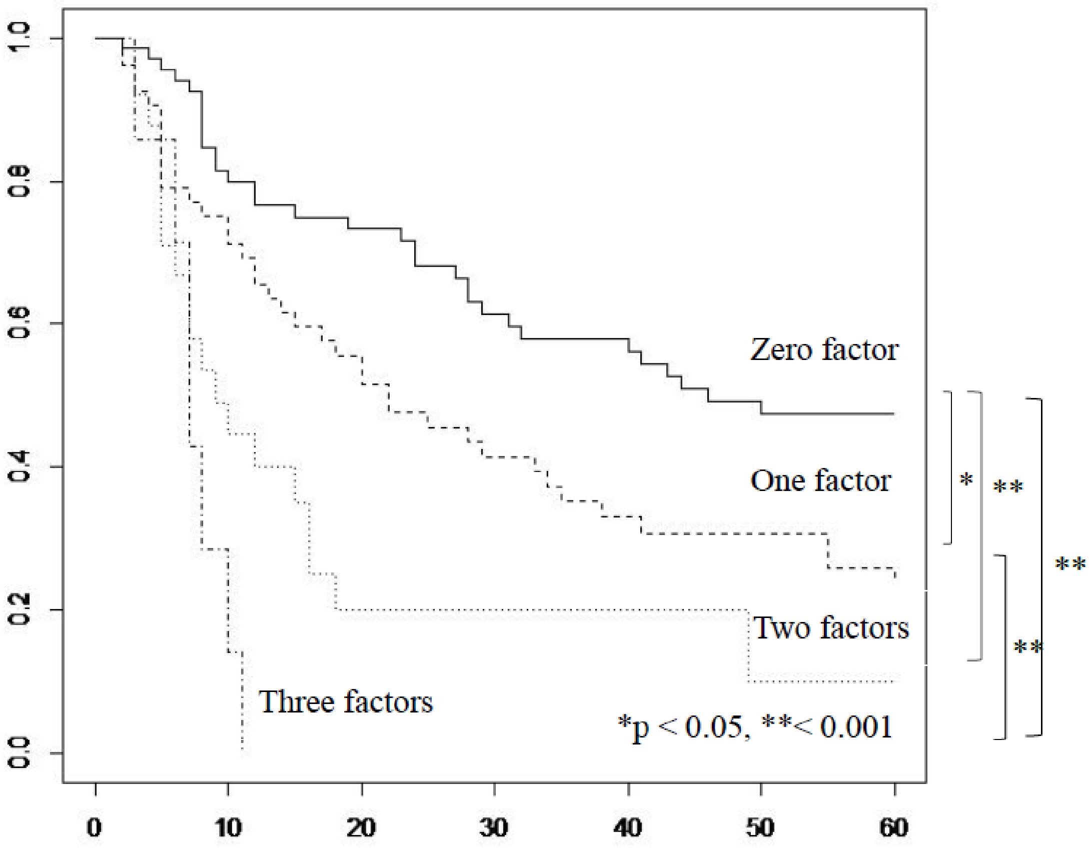

Figure S2. Recurrence-free survival (RFS) rate depending on the number of prognostic factors in patients with stage 3/4 gallbladder cancer without distant metastasis.

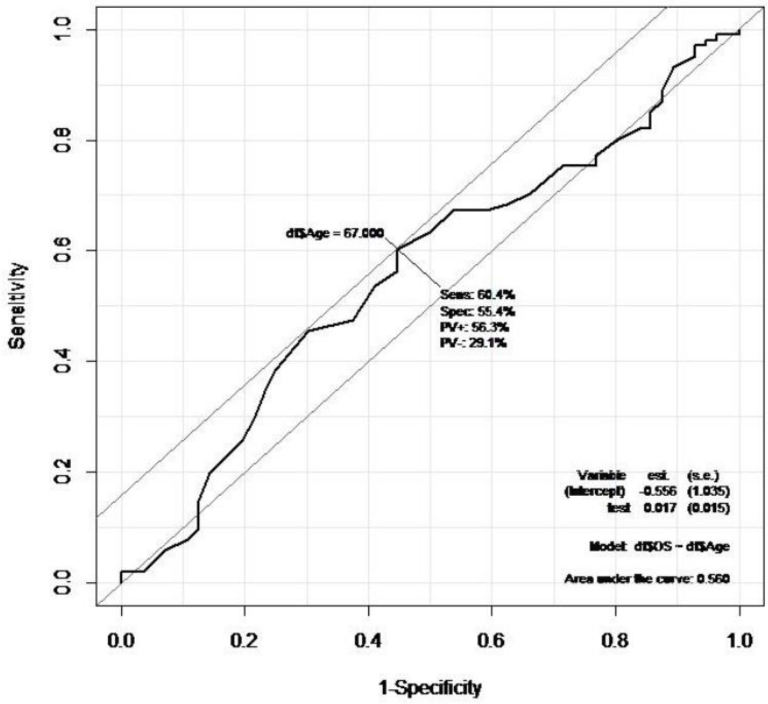
1.0
0.8
0.6
0.4
0.2
0.0
Sensitivity
dt$Age = 67.000
Sens: 60.4%
Spec: 55.4%
PV+: 56.3%
PV-: 29.1%
Varinble est. (s.e.)
(Intercept) -0.556 (1.035)
test 0.017 (0.015)
Model: dt$OS ~ dt$Age
Area under the curve: 0.560
0.0
0.2
0.4
0.6
0.8
1.0
1-Specificity

(**A**)

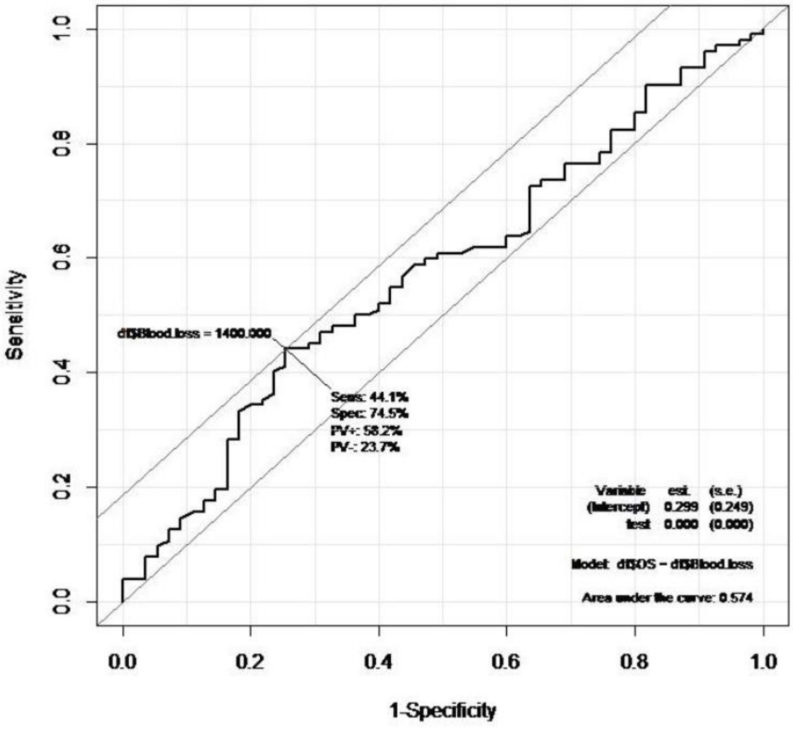
1.0
0.8
0.6
0.4
0.2
0.0
Sensitivity
dt$Blood.loss = 1400.000
Sens: 44.1%
Spec: 74.5%
PV+: 58.2%
PV-: 23.7%
Varinble est. (s.e.)
(Intercept) 0.299 (0.249)
test 0.000 (0.000)
Model: dt$OS ~ dt$Blood.loss
Area under the curve: 0.574
0.0
0.2
0.4
0.6
0.8
1.0
1-Specificity

(**B**)

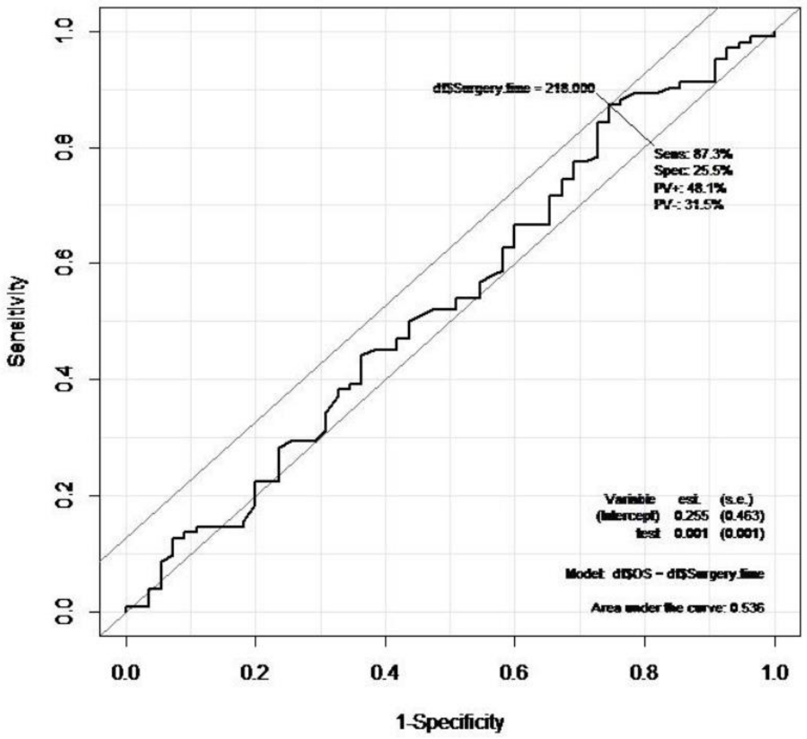

(**C**)

Figure S3. (**A**)The receiver operating characteristic analysis of age with death used as an event. PV-: negative predictive value; PV+: positive predictive value; Sens: sensitivity; Spec: specificity. (**B**)The receiver operating characteristic analysis of blood loss with death used as an event. PV-: negative predictive value; PV+: positive predictive value; Sens: sensitivity; Spec: specificity. (**C**)The receiver operating characteristic analysis of surgery time with death used as an event. PV-: negative predictive value; PV+: positive predictive value; Sens: sensitivity; Spec: specificity.

© 2020 by the authors. Licensee MDPI, Basel, Switzerland. This article is an open access article distributed under the terms and conditions of the Creative Commons Attribution (CC BY) license (http://creativecommons.org/licenses/by/4.0/).
